# Development and Validation of a LC-ESI-MS/MS Method for the Determination of *Alternaria* Toxins Alternariol, Alternariol Methyl-Ether and Tentoxin in Tomato and Tomato-Based Products

**DOI:** 10.3390/toxins8110328

**Published:** 2016-11-11

**Authors:** Yelko Rodríguez-Carrasco, Jordi Mañes, Houda Berrada, Cristina Juan

**Affiliations:** Department of Food Chemistry and Toxicology, Faculty of Pharmacy, University of Valencia, Av. Vicent Andrés Estellés s/n, 46100 Burjassot, Spain; jordi.manes@uv.es (J.M.); houda.berrada@uv.es (H.B.); cristina.juan@uv.es (C.J.)

**Keywords:** *Alternaria*, LC-MS/MS, dispersive liquid-liquid microextraction, tomato

## Abstract

*Alternaria* species are capable of producing several secondary toxic metabolites in infected plants and in agricultural commodities, which play important roles in food safety. *Alternaria alternata* turn out to be the most frequent fungal species invading tomatoes. Alternariol (AOH), alternariol monomethyl ether (AME), and tentoxin (TEN) are some of the main *Alternaria* mycotoxins that can be found as contaminants in food. In this work, an analytical method based on liquid chromatography (LC) tandem mass spectrometry (MS/MS) detection for the simultaneous quantification of AOH, AME, and TEN in tomato and tomato-based products was developed. Mycotoxin analysis was performed by dispersive liquid-liquid microextraction (DLLME) combined with LC-ESI-MS/MS. Careful optimization of the MS/MS parameters was performed with an LC/MS system with the ESI interface in the positive ion mode. Mycotoxins were efficiently extracted from sample extract into a droplet of chloroform (100 µL) by DLLME technique using acetonitrile as a disperser solvent. Method validation following the Commission Decision No. 2002/657/EC was carried out by using tomato juice as a blank matrix. Limits of detection and quantitation were, respectively, in the range 0.7 and 3.5 ng/g. Recovery rates were above 80%. Relative standard deviations of repeatability (RSDr) and intermediate reproducibility (RSD_R_) were ≤ 9% and ≤ 15%, respectively, at levels of 25 and 50 ng/g. Five out of 30 analyzed samples resulted positive to at least one *Alternaria* toxin investigated. AOH was the most common *Alternaria* toxin found, but at levels close to LOQ (average content: 3.75 ng/g).

## 1. Introduction

*Alternaria* is a widely distributed fungal genus frequently isolated from different plant crops, and has been documented as a pre- and post-harvest pathogen causing decay [1]. The fungal species of *Alternaria* are considered relevant contaminants of refrigerated fruits, vegetables, and stored foodstuffs, mainly as a consequence of their occurrence and the ability to grow and produce toxins even at low temperatures and low water activity [2]. *Alternaria* species produce a liarge variety of secondary metabolites capable of causing several health problems in humans and animals. The most relevant mycotoxins produced by *Alternaria* spp. are alternariol (AOH), alternariol monomethyl ether (AME), tentoxin (TEN), tenuazonic acid (TeA), altenuene (ALT), and altertoxins (ATXs).

The toxic effects of *Alternaria* toxins are wide-ranging. To date some of these mycotoxins have shown to be teratogenic in vivo. Genotoxic effects of AOH and AME in vitro have also been described [3]. Recently, some authors reported that AOH and AME are able to induce cell cycle arrest, apoptosis of cells, and DNA damaging effects [4,5,6,7]. In spite of the before-mentioned, there are currently no guideline limits set for *Alternaria* mycotoxins by regulatory authorities yet. The European Food Safety Agency (EFSA) provided a scientific opinion on the risks for animal and public health related to the presence of *Alternaria* toxins in feed and food [3]. EFSA evidenced a lack of robust occurrence data of *Alternaria* toxins in food and processed products, and recommended the collection of representative data across Europe to enable a proper risk assessment.

Tomatoes and many other soft-skinned vegetables and fruits can be easily infected by fungi and *Alternaria* is the main fungus responsible for spoilage. Tomato (*Solanum lycopersicum* L., syn. *Lycopersicon esculentum* Mill.) is considered to be one of the main important vegetable crops worldwide. Although tomatoes are commonly consumed fresh, over 80% of the tomato consumption comes from processed products, such as tomato juice, paste, puree, ketchup, and soup, such as gazpacho, a traditional Spanish, ready-to-serve cold vegetable soup, which contain fresh tomato (> 50%) and other ingredients, such as cucumber, pepper, olive oil, and other minor constituents, such as onion, garlic, wine vinegar, salt, and water.

Based on the increasing need for incidence data, a bunch of new analytical methods are demanded for detection and quantification of *Alternaria* toxins in foods. The coupling of both liquid (LC) and gas chromatography (GC) to tandem mass spectrometry (MS/MS) has enabled the development of highly selective, sensitive, and accurate methods for mycotoxin determination in both biological [8,9] and food samples [10,11]. For the analysis of mycotoxins in various food matrices, the traditional liquid-liquid extraction (LLE), solid phase extraction (SPE), and combinations of LLE and SPE have been commonly used as sample preparation procedures as recently reviewed by Turner et al. [12]. An ideal sample preparation procedure should be straightforward and rapid with low operational cost, as well as efficient in sample clean-up. Furthermore, to allow the trace-analysis of various compounds, a high enrichment factor could be of interest. To fulfill these ideal requirements, in 2006 it was proposed a novel very attractive dispersive liquid-liquid microextraction technique (DLLME) for the treatment of liquid samples, and has been recently used in the determination of some mycotoxins in several food samples [13,14,15,16,17]. Basically, DLLME consists of the formation of a cloudy solution promoted by the fast addition of a mixture of extraction and disperser solvents to an aqueous sample. The tiny droplets formed and dispersed among the aqueous sample solution are further joined by centrifugation. DLLME has been proved to be a powerful cleaning and preconcentration technique. Other benefits are its high speeds, the low solvent use, and final disposal.

The objective of the present work was to develop and validate a reliable DLLME-LC-ESI-MS/MS method for simultaneous determination of some *Alternaria* toxins in tomato and tomato-based products. Special attention was given on the optimization of the MS/MS parameters to attain the best response. Additionally, optimization of the DLLME procedure was also assessed, by careful evaluation of the nature and amount of extraction and disperser solvents as well as the amount of sample. The validated method was used to assess the occurrence of AOH, AME, and TEN in 30 tomato and tomato-based samples commercialized in Valencia, Spain.

## 2. Results and Discussion

### 2.1. MS/MS Optimization

A preliminary study was conducted in order to obtain the best instrumental conditions affording high resolution and short analysis time with a suitable analyte separation. The optimization of the analyte-dependent MS/MS parameters was performed via direct infusion of standards (diluted in a 1:1 mixture of eluent A and B) into the MS source using a syringe injection at a flow rate of 10 µL/min. Positive and negative ionization modes were tested, obtaining a better response in positive ionization mode for the studied *Alternaria* toxins. Compound-dependent parameters of quadrupole mode scans including declustering potential (DP), entrance potential (EP), collision cell entrance potential (CEP), collision cell exit potential (CXP), and collision energies (CE) were also evaluated and optimized to provide the best combination of efficiency and finding the optimal response value for each analyte. The acquisition of two single reaction monitoring transitions per analyte allowed the confirmation of the identity of the positive results according to the criteria established in Commission Decision No. 2002/657/EC [18]. The product ion with the highest intensity was selected as a quantifier, whereas the other was used as a qualifier. Table 1 lists the characteristic ions and the optimized mass spectrometry parameters for each compound during multiple reaction monitoring (MRM) acquisitions.

### 2.2. DLLME Optimization

DLLME has been proved to be a powerful cleaning and preconcentration technique. Other benefits, such as its high speeds and the low solvent use (if compared with traditional sample preparation procedures) and final disposal, have caused it to fast become one of the most popular analytical sample preparations in developing reliable quantitative multi-analyte methods. Other promising techniques, such as dilute-and-shoot approaches, only requires the dilution of the sample, however, it does not remove any interference which can cause chromatographic troubles, such as carryover, loss of sensitivity, increase of background interferences, or co-eluting peaks. Therefore, its use as a routine procedure may cause a time consuming drawback coming from the troubleshooting and cleaning of the system.

Hence, the DLLME sample preparation procedure was selected here based on its benefit in routine on a daily basis. The effect of the following parameters affecting the extraction efficiency was evaluated: (i) the type of extraction and disperser solvents; (ii) the extraction-disperser solvent ratios; and (iii) the amount of sample. The method of optimization was performed by recovery experiments in three replicates using tomato extract blank samples (5 mL) spiked with 25 ng/g of each targeted mycotoxin.

#### 2.2.1. Influence of the Type of Extraction and Disperser Solvents

Selection of both an appropriate extraction and disperser solvent is very crucial to achieve good performance. The extraction solvent must have properties, such as a greater density than water, high extraction capability of the analytes, as well as low solubility in water. The role of the disperser solvent is to increase the dispersion of the extraction solvent as tiny droplets in an aqueous medium solution resulting in a large contact area between the extraction solvent and aqueous solution, thus improving the extraction efficiency. In this study, three common halogenated solvents, including CCl_4_, CH_2_Cl_2_, and CHCl_3_ were tested for extraction, whereas acetonitrile, acetone, and methanol were selected as disperser solvents.

Mixtures of 1.0 mL of different disperser solvents and 100 μL of extraction solvent were injected to 5 mL of tomato sample extracts spiked with the standard solution at 50 ng/g. Furthermore, 1 g of NaCl was added to the sample extract to improve the extraction efficiency, as well as to facilitate the phase’s separation [19]. Extraction efficiency was evaluated by comparing the recoveries of the analytes. Results indicated that the best conditions were accomplished with the AcN-CHCl_3_ pair, with satisfactory recoveries between 81%–94% (Table 2). There were no differences in extraction for the studied analytes.

#### 2.2.2. Influence of the Extraction-Disperser Solvent Ratio

**Figure 1:**
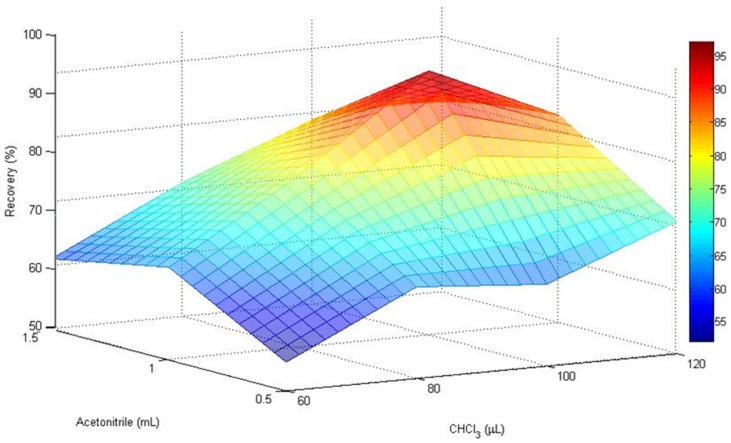
MATLAB-based surface response design showing the influence of AcN and CHCl_3_ ratio in the extraction efficiency of AOH. Recovery experiments were conducted by spiking tomato extract blank samples with 25 ng/g of each targeted mycotoxin.

To evaluate the influence of the extraction-disperser solvent ratios on the extraction efficiency different volumes of chloroform (60, 80, 100, and 120 µL) and acetonitrile (0.5, 1.0, and 1.5 mL) were used. The optimal volumes of AcN and CHCl_3_ were evaluated with the MATrix LABoratory (MATLAB)-based surface response design (Figure 1).

The enrichment factor improved with the lower volumes, but the lower the volumes the lesser the volume of the sedimented phase. Despite this, when the volume of extraction solvent was increased from 60 to 100 µL, the recoveries of the mycotoxins rose significantly. However, it should be noted that with the following combinations of AcN-CHCl_3_: 1.50 mL–120 µL, 1.50 mL–100 µL, and 1 mL–120 µL, the matrix effect increased with respect to the other tested ratios. Hence, the combination of 1 mL of acetonitrile containing 100 µL of CHCl_3_ was selected as a good compromise to reach the best DLLME conditions.

#### 2.2.3. Influence of the Amount of Sample

**Figure 2:**
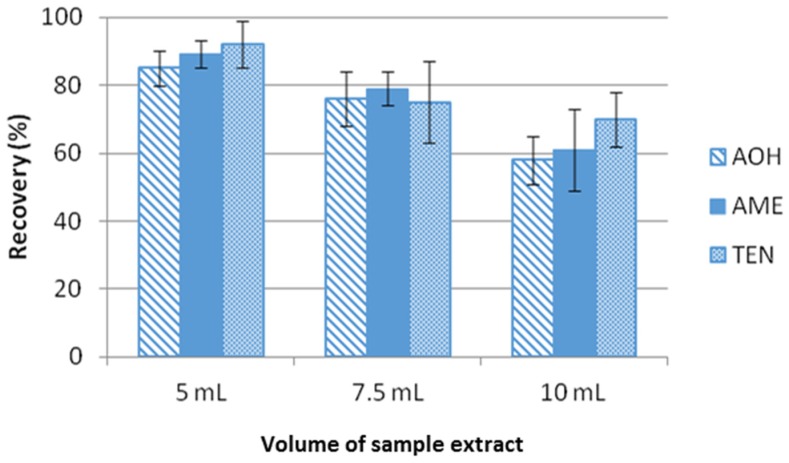
Influence of the volume of sample extract in the extraction efficiency of the *Alternaria* toxins studied. Recovery experiments were conducted by spiking tomato extract blank samples with 25 ng/g of each target mycotoxin.

The influence of the amount of sample was evaluated by testing different amounts of sample extract (5, 7.5 and 10 mL). Results showed that recoveries below 80% were obtained with 7.5 and 10 mL of sample, whereas recoveries greater than 80% were achieved with 5 mL. Thus, 5 mL of tomato extract was selected as the optimum amount of sample for a reliable and efficient extraction (Figure 2).

### 2.3. Analytical Method Validation

Good linearity was achieved in all cases with regression coefficients higher than 0.990. Significant signal suppression was observed (from 65%–80%) between the slopes of the calibration lines meaning that the matrix effect is present (Table 3). Therefore, matrix-matched calibration curves were used for effective quantification in tomato samples.

Satisfactory results in terms of recoveries were found (recovery range from 81%–94% for both spiking levels). Precision studies showed that the method was repeatable (RSD_r_ < 9%) and reproducible (RSD_R_ < 15%) (Table 3).

**Figure 3:**
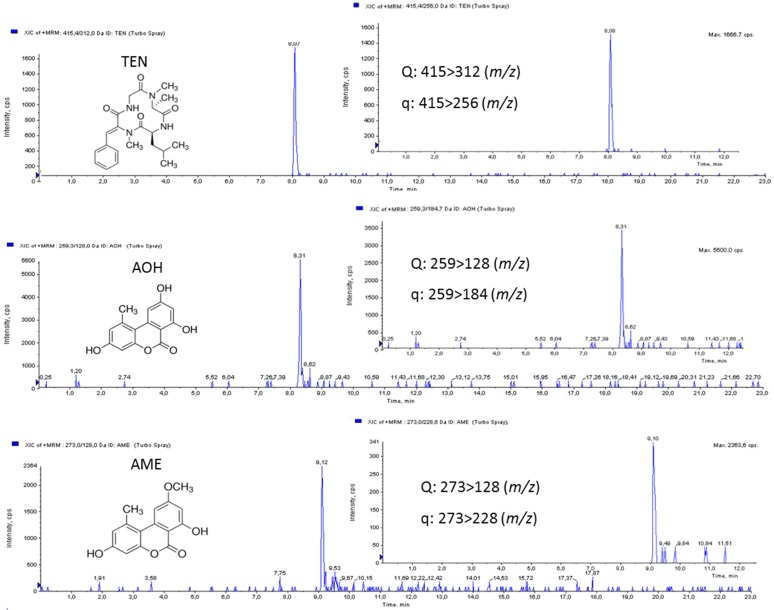
MRM chromatograms of tomato juice spiked with 3.5 µg/L of AOH, AME, and TEN (corresponding to 1 ng of each injected toxin).

LOQs were 3.5 ng/g for AOH and AME, while the LOQ for TEN was 1.75 ng/g. LODs were 1.40 ng/g for AOH and AME, while the LOD for TEN was 0.70 ng/g (Table 3). These results showed the suitability of the developed method for the determination of trace amounts of the selected mycotoxins in tomato samples. No obvious interfering peak from blank samples was detected. MRM chromatograms of tomato juice spiked at 10 µg/L of AOH, AME, and TEN are shown in Figure 3.

The results obtained in the present study were within the limits set by Commission Decision, No. 2002/657/EC. According to Commission Decision, No. 2002/657/EC, it is acceptable that trueness of measurements is assessed through recovery of additions of known amounts of the analytes to a blank matrix and the guideline ranges for the deviation of the experimentally-determined recovery should be between 80% and 110% for a mass fraction ≥ 10 µg/kg. In the case of repeated analysis of a sample carried out under within-laboratory reproducibility conditions, the intra-laboratory relative standard deviation should not exceed 20% for a mass fraction of ≥ 10 µg/kg to 100 µg/kg. For analyses carried out under within-laboratory reproducibility conditions, the within-laboratory RSD shall not be greater than the reproducibility RSD.

### 2.4. Application to Samples

**Figure 4:**
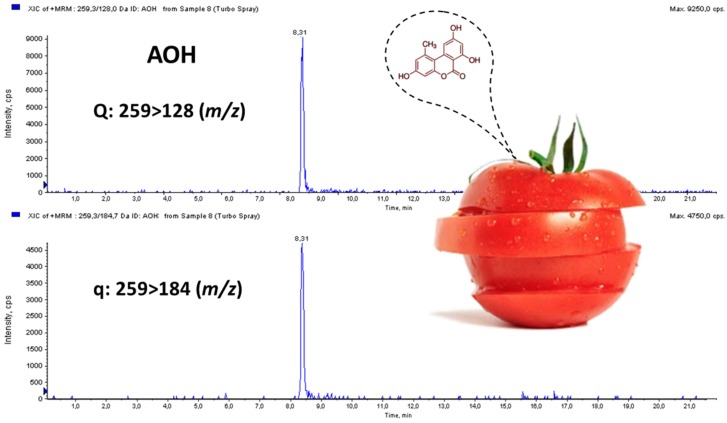
MRM chromatogram of a naturally contaminated tomato sample with AOH at 5.8 ng/g.

The developed method was evaluated carrying out a survey of AOH, AME, and TEN in 30 tomato and tomato-based products purchased in several Valencian supermarkets (Spain). Neither in tomato juice (*n =* 5) nor in gazpacho (*n =* 5) samples was the occurrence of target mycotoxins detected. However, five out of 20 fresh tomato samples (25%) resulted positive to at least one *Alternaria* toxin. AOH was detected in four out of the five contaminated samples but at levels close to LOQ (mean: 3.75 ng/g), whereas AME was identified in two fresh tomato samples but at levels between LOD and LOQ. TEN was not found in any analyzed sample. A MRM chromatogram of a naturally-contaminated tomato sample with AOH at 5.8 ng/g is shown in Figure 4.

These findings are in agreement with the results reported in some European studies. In a study conducted in The Netherlands, AOH was detected in three out of 10 fresh tomato samples (levels ranging between 2–15 ng/g) but AME and TEN were not detected [20]. However, in the same study, AOH was quantified (at levels from 2–11 ng/g) in four out of 14 tomato juice samples. Similarly, in Switzerland, no *Alternaria* mycotoxins were found in fresh and whole tomato samples (*n =* 4) but AOH was detected in eight out of 24 tomato soup samples (levels from 4–10 ng/g). In the same products AME was detected in seven out of 24 samples but at lower concentration (from 1–4 ng/g). Only a few samples were positive for TEN (tomato puree, concentrated, and dried tomatoes) [21]. Those findings are also in line with the results reported in Italian tomato-based products (*n =* 10). Occurrence of AOH was detected in five out of 10 samples (levels from 4–6.8 ng/g) and TEN in one sample (4.4 ng/g) whereas AME was not found [22]. In Germany, a higher occurrence of *Alternaria* mycotoxins in tomato products (*n =* 34) was recently reported [23]. AME, AOH, and TEN were detected in 100, 70, and 26% of samples, respectively. AOH was found at levels from 6.1–25 ng/g and AME from 1.2–7.4 ng/g, whereas TEN contamination was set at levels from LOD and LOQ (<6.6 ng/g).

In China, no *Alternaria* mycotoxins were found in a study conducted in 70 fresh tomato samples [24]. In contrast, contamination of 26.2% of AME (up to 1734 µg/kg) and 6.2% of AOH (up to 8756 µg/kg) was reported in 80 tomato purees processed and sold in Argentina [25]. Similar results were also reported by Van de Perre et al. [26] who analyzed a total of 144 samples of derived tomato products such as ketchups, concentrates, pulp, dried tomatoes, and juices, which were collected from local markets in different countries (i.e., Belgium, Spain, Egypt, Brazil, and South Africa). Puree and concentrate tomato samples showed the highest occurrence of AOH and AME whereas, in tomato juice samples, none of the studied toxins were detected.

## 3. Conclusions

A rapid, straight-forward, robust, sensitive and accurate analytical method based on DLLME-LC-ESI-MS/MS for determining various *Alternaria* toxins in tomato and tomato-based products was developed. Careful optimization of the MS/MS parameters was performed to reach the best analytical conditions. Additionally, parameters affecting the extraction efficiency of the DLLME were also evaluated and optimized. The method performance fulfilled the EU guideline standardized in the Commission Decision, No. 2002/657/EC. The recoveries were greater than 80% and relative standard deviations of repeatability and intermediate reproducibility were ≤9% and ≤15%, respectively, at levels of 25 and 50 ng/g. Under the optimized conditions LODs and LOQs were in the range 0.7–3.5 ng/g, respectively. Significant signal suppression was observed and matrix-matched calibrations were used for quantitation purpose. The developed method was successfully applied to 30 commercially available tomato and tomato-based products acquired in Valencia, showing the occurrence of various *Alternaria* toxins, at levels of few nanograms per gram in 20% of samples, being AOH the most commonly mycotoxin found. Due to its simplicity, and by allowing a faster extraction, the proposed methodology is proposed as a reliable analytical tool. Furthermore, this method could be applied to gather data on the presence of *Alternaria* toxins in foodstuffs, which are highly recommended by EFSA to enable a proper risk assessment of these toxins.

## 4. Materials and Methods

### 4.1. Chemicals and Reagents

Acetonitrile (AcN), methanol (MeOH), acetone (Ac), chloroform (CHCl_3_), dichloromethane (CH_2_Cl_2_), and carbone tetrachloride (CCl_4_) were supplied by Merck (Darmstadt, Germany). Ammonium formate (99%) was supplied by Panreac Quimica S.A.U. (Barcelona, Spain). Deionized water was obtained in the laboratory using a Milli-Q SP^®^ Reagent Water System (Millipore, Bedford, MA, USA).

Certified standards of AOH, AME and TEN were purchased from Sigma-Aldrich (Madrid, Spain). Standard solutions of AOH, AME, and TEN were prepared by dissolving 10 mg of each compound in 10 mL of MeOH. Stock solutions were diluted with pure MeOH afterwards order to get the appropriate working solutions. A multi-mycotoxin working standard solution was prepared by combining aliquots of each individual working solution and diluting with MeOH to obtain the final concentration of 0.02 mg/L for AOH, AME, and TEN. All solutions were stored at −20 °C in amber glass vials and darkness before use.

### 4.2. LC-MS/MS Analysis

The determination was performed using a system LC-MS/MS triple quadrupole, consisted of a LC Agilent 1200 (Agilent Technologies, Santa Clara, CA, USA)using a binary pump and automatic injector and coupled to a 3200 QTRAP^®^ AB SCIEX (Applied Biosystems, Foster City, CA, USA). The chromatographic separation of the analyte was conducted at 25 °C with a reverse phase analytical column Gemini^®^ C18 (3 μM, 150 × 2 mm ID) and a guard-column C18 (4 × 2 mm, ID; 3 μM) from Phenomenex (Madrid, Spain).

Mobile phase was a time programmed gradient using as phase A methanol (1% formic acid and 5 mM ammonium formate), and as phase B water (1% formic acid and 5 mM ammonium formate). The following gradient was employed: equilibration during 2 min at 10% A at 0.25 mL/min, 10%–80% A in 3 min at 0.25 mL/min, 80% A for 1 min at 0.25 mL/min, 80%–90% A in 2 min, 90% A for 6 min at 0.25 mL/min, 90%–100% A in 3 min at 0.25 mL/min, 100% for 1 min at 0.35 mL/min, 100%–50% in 3 min at 0.4 mL/min at, return to initial conditions in 2 min and maintain during 2 min. Total run time was 21 min. The injection volume was 20 µL.

To analyze the mycotoxins, a triple quadrupole mass spectrometry detector (MS/MS) 3200 QTRAP^®^ System AB SCIEX (Applied Biosystems, Concord, ON, Canada) was used. Electrospray ionization (ESI) interfaces were used to analyze these mycotoxins with the following settings for source/gas parameters: curtain gas (CUR) 20, ionspray voltage (IS) 5500 V, source temperature (TEM) 450 °C, ion source gas 1 (GS1), and ion source gas 2 (GS2) 50. Therefore, in this study, the optimization of the MS/MS parameters was performed with an LC/MS system with the ESI interface in the positive ion mode using a mycotoxin standard mixture. The precursor ions (Q1) of each mycotoxin were confirmed in product ion (Q3) scan mode. As shown in Table 1, a protonated molecule was observed as the base peak ion in the mass spectra of AOH, AME, and TEN. Hence, these ions were selected as precursor ions (Q1) for each mycotoxin. The optimization of product ions (Q3) and their collision energy were performed in the product ion scan mode. The final selection of multiple reaction monitoring (MRM) transitions in positive ion mode for each compound, the optimal declustering potential (DP), entrance potential (EP), collision cell entrance potential (CEP), collision cell exit potential (CXP), and collision energies (CE) are shown in Table 1. Data acquisition and processing were performed using Analyst^®^ software version 1.5.2. (MDS Analytical Technologies, 2008 MDS Inc, ON, Canada,).

### 4.3. Sampling and Sample Preparation

Thirty tomato and tomato-based samples were purchased from local supermarkets located in Valencia (Spain). Unwashed fresh tomato samples (*n =* 20) were chopped using a blender immediately after reception of samples in the laboratory. After blending, homogenization, and centrifugation (3000 rpm, 4° C for 5 min), the tomato samples were placed in 100 mL closed polyethylene flasks before storing at 4 °C until analysis. Analyses were carried out within the following two days after reception. Tomato-based samples consisted of gazpacho samples (*n =* 5) and tomato juice (*n =* 5).

A modified version of the DLLME method for sample preparation of fruit juices was used [27]. A mixture of 1 mL of AcN (as disperser solvent) and 100 µL of CHCl_3_ (as extraction solvent), was rapidly injected into 5 mL of centrifuged tomato extract containing 1 g of NaCl. The mixture was vortexed for 1 min and centrifuged at 4000 rpm for 5 min, and the droplet formed was collected by a 100 µL syringe and transferred to a chromatography vial. Then, the droplet was evaporated to dryness under a gentle stream of N_2_ and reconstituted with 1 mL of MeOH:H_2_O (50:50, *v/v*). The solution was filtered through 13 mm/0.20 µm nylon filter and injected into the LC–MS/MS system for mycotoxin analysis.

### 4.4. Method Performance

The method performance was performed under optimized conditions following the Commission Decision No. 2002/657/EC. The method validation included the evaluation of linearity, limits of detection (LODs), limits of quantification (LOQs), recoveries, repeatability (intra-day precision), and intermediate reproducibility (inter-day precision). All of the parameters were evaluated by spiking blank tomato juice samples at 25 and 50 ng/g. Samples were spiked and left to equilibrate over night before the analysis.

Linearity was assessed through six concentration levels in a linear range between LOQ and 100 × LOQ in triplicate. The correlation coefficient was obtained by plotting the signal intensity against analyte concentrations. A calibration curve was injected at the end of each batch to assess the response drift of the method. Components from matrix can negatively influence the method performance if they co-elute with the analyte of interest and can cause ion suppression or enhancement in the ion source. Therefore, matrix effect was also evaluated. The matrix effect (ME), is defined as the ratio between the slopes of the matrix-matched calibration and the solvent calibration one, and it was calculated as follows:

ME(%)=Slopematrix-matchedSlopesolvent ×100

Matrix-matched calibration curves were built by spiking blank sample extracts with the studied analytes at the same concentration levels than those used in solvent standard calibration curves.

The accuracy was evaluated through recovery studies using spiked blank samples at 25 ng/g and 50 ng/g concentration levels. Recovery studies were performed in triplicate in the same day, as well as in three different days. Precision (expressed as %RSD) of the method was determined by repeatability (intraday precision, RSDr) and intermediate reproducibility (interday precision, RSDR). Intraday variation was evaluated in three determinations per concentration in a single day, whereas interday variation was tested on three different working days within 20 days. RSDr and RSDR were determined by spiking blank samples at the 25 ng/g and 50 ng/g concentration levels.

Limits of detection (LODs) and limits of quantitation (LOQs) were estimated from a blank juice tomato sample fortified with decreasing concentrations of the analytes. LODs were calculated using a signal-to-noise ratio of 3:1. LOQs Results were calculated using a signal-to-noise ratio of 10:1. The specificity of the method was evaluated with respect to interferences from endogenous compounds. Five samples of blank tomato juice samples were analyzed and compared with the corresponding spiked samples at the LOQ level to check for possible interference with the detection of the analytes.

### 4.5. Confirmation Criteria

Confirmation criteria were based on the following items; (i) chromatographic separation: the retention time of the analyte in the extract should correspond to that of the matrix-matched calibration within a ± 2.5% interval of the retention time; (ii) mass spectrometric detection: extracted ion chromatograms of sample extracts should have peak shapes and response ratios to those obtained from calibration standards analyzed at comparable concentrations in the same batch. The relative intensities or ratios of selective ions, expressed as a ratio relative to the most intense ion used for identification, should correspond to those of the calibration standard solutions. The ion ratio should not deviate more than 30% (relative).

## Figures and Tables

**Table 1:** Retention times, main transitions, collision energies (CE), declustering potential (DP), entrance potential (EP), collision cell entrance potential (CEP), and collision cell exit potential (CXP) for the *Alternaria* toxins analyzed in this study.

Analyte	Rt (min)	Parent Ion Q1 (m/z)	Product Ion Q3 (m/z)	CE (V)	DP (V)	EP (V)	CEP (V)	CXP (V)
TEN	8.1	415	312 ^Q^	29	55	8	21	2
		[M + H]^+^	256 ^q^	39				
AOH	8.3	259	128 ^Q^	65	39	10	16	3
		[M + H]^+^	184 ^q^	42				
AME	9.1	273	128 ^Q^	60	32	10	16	13
		[M + H]^+^	228 ^q^	40				

^Q^ Quantification transition; ^q^ Qualification transition.

**Table 2:** Recovery range of *Alternaria* toxins obtained by using different combinations of extraction (Ac, AcN and MeOH) and disperser solvents (CCl_4_, CH_2_Cl_2_, and CHCl_3_).

Disperser Solvent	Recovery Range (%) ^a^		
	Extraction Solvent		
	CCl_4_	CH_2_Cl_2_	CHCl_3_
Ac	45–67	35–56	69–78
AcN	71–86	47–66	81–94
MeOH	58–81	53–78	65–83

^a^ spiked level: 25 ng/g of each target mycotoxin.

**Table 3:** Overview of the correlation coefficient, extraction recovery, repeatability, and reproducibility (Rec (RSD), %), limits of detection (LODs) and quantitation (LOQ), and signal suppression/enhancement (SSE) for the studied analytes.

Mycotoxin	Correlation Coefficient (r)	Repeatability (RSD_r_, %) ^a^		Reproducibility (RSD_R_, %) ^a^		LOD (ng/g)	LOQ (ng/g)	SSE (%)
		25 ng/g ^b^	50 ng/g ^b^	25 ng/g ^b^	50 ng/g ^b^			
AOH	0.998	81 (6)	82 (4)	84 (8)	89 (6)	1.40	3.50	65
AME	0.996	86 (4)	89 (7)	90 (7)	93 (10)	1.40	3.50	80
TEN	0.995	91 (9)	94 (6)	94 (15)	90 (12)	0.70	1.75	78

^a^ *n =* 3; ^b^ spiked level.
